# Evaluation of non‐invasive prenatal testing to detect chromosomal aberrations in a Chinese cohort

**DOI:** 10.1111/jcmm.14614

**Published:** 2019-08-27

**Authors:** Wanting Cui, Xiaoliang Liu, Yuanyuan Zhang, Yueping Wang, Guoming Chu, Rong He, Yanyan Zhao

**Affiliations:** ^1^ Shengjing Hospital of China Medical University Shenyang China

**Keywords:** copy number variations, genetic counselling, non‐invasive prenatal testing, prenatal diagnosis

## Abstract

The aim of this study was to evaluate the clinical feasibility of non‐invasive prenatal testing (NIPT) to detect foetal copy number variations (CNVs). Next‐generation sequencing for detecting foetal copy number variations (CNVs) was performed on the collected samples from 161 pregnancies with ultrasound anomalies and negative NIPT results for aneuploidy. The performance of NIPT for detecting chromosome aberrations was calculated. The sensitivity and specificity of NIPT for detecting CNVs > 1 Mb were 83.33% and 99.34%; the PPV and negative predictive rate (NPV) were 90.91% and 98.68%. Non‐invasive prenatal testing can be performed to detect chromosomal aberrations in first trimester with high performance for CNVs, and occasional discordant cases are unavoidable.

## 1 INTRODUCTION

Non‐invasive prenatal testing (NIPT) with next‐generation sequencing (NGS) on cell‐free foetal DNA (cffDNA) in maternal plasma has been widely used for foetal aneuploidy screening because it can reduce unnecessary invasive procedures that may result in miscarriage or intrauterine infection.^1^ Traditional prenatal diagnosis strategies have been altered since NIPT is considered as a primary prenatal screening method due to the high accuracy.^2^, ^3^, ^4^ Copy number variation sequencing (CNV‐seq) with higher resolution has been applied to diagnose foetal submicroscopic chromosome abnormalities invasively.^5^, ^6^ Accordingly, non‐invasive detection of foetal CNVs was encouraged by the successful application of NGS on cffDNA in screening for aneuploidy. Li et al^7^ and Liu et al^8^ illustrated that NIPT exhibited higher performance in detecting large CNVs, and the detection power for smaller CNV sizes decreased when the same sequencing depth was used to detect aneuploidy. Yin et al^9^ and Lo et al 10illustrated the variation in detection power among CNVs of the same size, but at different sequencing depths and foetal fractions. Current researches suggest that the resolution of CNVs detected by NIPT can reach 1Mb with present technologies, and the accuracy in detecting CNVs > 10 Mb is sufficiently high. The latest large cohort study by Liang et al^11^ documented that non‐invasive screening for CNVs can be adopted as the first‐tier prenatal approach. But the controversy on the utility of NIPT for detecting foetal CNVs is still remained,^12^ and more evidences are needed to explore the clinical feasibility. In our study, we evaluated the screening effectiveness of NIPT to detect CNVs in pregnancies with abnormal ultrasound findings, which are common indications for invasive genetic testing.

## 2 MATERIALS AND METHODS

### 2.1 Study design

We gathered the pregnancies with ultrasound anomalies and negative NIPT results for aneuploidy. Maternal blood samples and foetal samples such as amniotic fluid or foetal tissues were collected. Non‐invasive prenatal testing was performed on maternal plasma for detecting foetal CNVs, and diagnosing CNVs in foetal samples was by CNV sequencing (CNV‐seq). The resolution of CNVs was no less than 1 Mb, and the pathogenicities of identified CNVs were evaluated following American College of Medical Genetics and Genomics (ACMG) guidelines. The karyotypes of foetuses and their parents were obtained by G‐banding karyotyping. All participants were offered genetic counselling and gave informed consent. This study was approved by the institutional review board of Shengjing Hospital.

### 2.2 Detecting foetal CNVs in maternal plasma with next‐generation sequencing (NGS)

Five mL of maternal peripheral blood was collected in an EDTA‐containing Vacutainer tube (Becton Dickenson) and centrifuged at 1600 g for 10 minutes at 4°C The plasma and white blood cells were transferred to microcentrifuge tubes separately, and the plasma was centrifuged again at 16 000 g for 10 minutes, then stored at −20°C. Plasma circulating cell‐free DNA (cfDNA) was extracted using the Circulating Nucleic Acid kit (Berry Genomics) from 700 μL of stored plasma. The concentration of cfDNA was quantitated using the Invitrogen Qubit 2.0 (ThermoFisher Scientific), with standards of 0.05‐0.7 ng/μL. Next, the cfDNA library was constructed using a non‐invasive prenatal test library prep kit (Berry Genomics), in which every sample was indexed by 6 bp indexing oligoes. Then, the cfDNA library was purified using the Purification DNA libraries for NGS kit from Berry Genomics. DNA libraries were quantitated using the Kapa SYBR fast qPCR kit (Kapa Biosystems), and the standard of DNA library concentrations was greater than 20 pmol/L. The quantitated DNA libraries were pooled and loaded into Illumina Nextseq CN500 flow cells (Illumina), then were sequenced using the single‐ended 36 bp sequencing protocol. No <10 million unique reads were analysed by the software provided by Berry Genomics. The minimum standard of foetal fraction was 4%.

### 2.3 CNV sequencing in foetal gDNA

Genomic DNA (gDNA) was extracted from amniotic fluid or foetal tissues using the Genomic DNA extraction kit (QIAGEN); then the gDNA was purified using the Purification DNA kit (Zymo Research). The concentration of gDNA was quantitated using the Invitrogen Qubit 2.0 (ThermoFisher Scientific), with the standard of greater than 8 ng/μL. The library was constructed and purified, using the same methods which were performed in maternal plasma. Then DNA libraries were quantitated using the Kapa SYBR fast qPCR kit from Kapa Biosystems, with the standard of greater than 25 nmol/L. The quantitated DNA libraries were pooled and loaded into Illumina Nextseq CN500 flow cells, then were sequenced using the single‐ended 36 bp sequencing protocol. No less than 2.5 million unique reads were analysed by the software provided by Berry Genomics.

### 2.4 Karyotyping

Amniotic fluid cells were cultured if amniocentesis was performed at 19‐24 gestational weeks. G‐banding karyotyping was performed on cultured amniotic fluid cells and peripheral blood obtained from the pregnant women and their partners. Six metaphase cells were analysed, and 20 metaphase cells were counted at a resolution of 350‐500 bands by two examiners who were double‐blinded. The karyotyping results were identified and described upon agreement of the two examiners, with reference to the International System for Human Cytogenetic Nomenclature 2016.

## 3 RESULTS

In 161 samples of maternal plasma, NIPT detected 11 CNVs ≥ 1 Mb in 9 samples, including two CNVs in each one of two separate samples. CNV‐seq was performed on 137 samples of amniotic fluid and 24 samples of foetal tissues, then 12 CNVs ≥ 1 Mb in 10 samples, including two CNVs in each one of two separate samples were detected. Foetal karyotypes were obtained in 78 cases, and 7 cases were diagnosed as abnormal.

**Figure 1:**
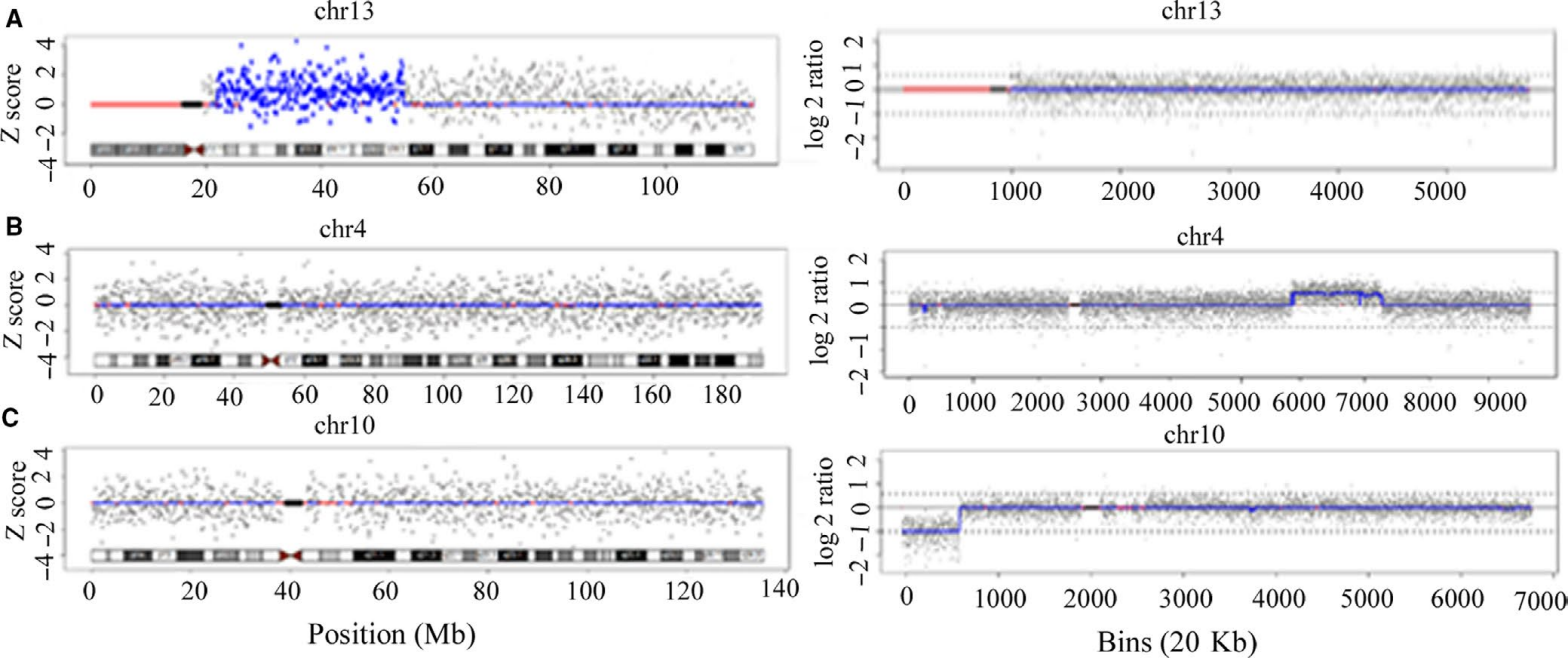
The comparative chromosome plots from NIPT (left) and CNV‐seq (right). A, In false positive case SJ023, NIPT detected a 32.5 Mb duplication at chr13 q12.11‐q14.3, which was not confirmed by CNV‐seq. B, In false negative case SJ015, CNV‐seq detected a 27.66 Mb duplication at chr4 q26‐q31.21, which was missed by NIPT. C, In false negative case SJ028, CNV‐seq detected a 12.29 Mb deletion at chr10 p15.3‐p13, which was missed by NIPT

By comparing the CNVs results in Table 1, a false positive case (SJ023) and two false negative cases (SJ015 and SJ028) were confirmed (Figure 1). Of the 10 true positive CNVs, the locations were coincident, but the sizes were slightly different. When the CNV‐seq results were compared with corresponding karyotypes, 2 CNVs (SJ047 and SJ022) did not appear to be direct duplication or deletion at telomeres in the karyotypes. A 3.4 Mb deletion should not have been detected by karyotyping in case SJ103. Through the available parental karyotypes, we were able to trace five CNVs from derivative chromosomes in 3 cases, and 5 de novo CNVs. As described in Table 2, the performance of NIPT for detecting CNVs was calculated depending on the standards of CNV‐seq results, achieving the sensitivity of 83.33%, the specificity of 99.34% and the PPV (positive predictive rate) of 90.91%. The sensitivity and specificity for CNVs between 1 Mb and 5 Mb were higher than those for CNVs ≥ 5 Mb. As shown in Table 1, among the 12 CNVs detected by CNV‐seq, 11 CNVs were pathogenic, and known CNVs syndromes were involved in 3 cases.

**Table 1:** Overview of 11 cases with detected CNVs

Cases	Ultrasound anomalies	NIPT results	CNV‐seq results	Karyotype	Pathogenicity	Concordance
SJ023	Hydroderma	chr13q12.11‐q14.3: (21900000‐54399999) X3;32.5 Mb	Negative	46,XY		False Positive
SJ015	VSD	Negative	chr4q26‐q31.21: (117480001‐145140000) X2.5;27.66 Mb	47,XX,+mar[23]/46,XX[21]	Uncertain	False Negative
SJ028	Increased NT	Negative	chr10p15.3‐p13: (148206‐12446689) X1;12.29 Mb	46,XY,del(10)(p16)	Pathogenic	False Negative
SJ027	Cystic hydroma; absence of nasal bone; VSD; renal dysplasia	chr4p16.3‐p15.2: (300000‐26099999) X1;25.8 Mb chr4q34.3‐q35.2: (178300000‐190599999) X3;12.3 Mb	chr4p16.3‐p15.2: (40001‐26080000) X1;26.04 Mb chr4q34.3‐q35.2: (178340001‐190360000) X3;12.02 Mb	46,XX, rec(4)dup(4q)inv(4) (p15.3q34)pat	Pathogenic, involved Wolf‐Hirschhorn Syndrome Pathogenic	True Positive
SJ036	VSD; AS	chr4q13.3‐q22.2: (71500001‐94900000) X1;23.4 Mb	chr4q13.3‐q22.3: (71240001‐97900000) X1;26.66 Mb	46,XY, der(4)t(4;13)(q22;q13)del(4)(q13q22)mat	Pathogenic	True Positive
SJ041	SUA; polyhydramnios	chr9p24.3‐p24.2: (190001‐4090000) X1;3.9 Mb chr11p15.5‐p15.1: (180001‐18080000) X3;17.9 Mb	chr9p24.3‐p24.2: (200001‐3920000) X1;3.72 Mb chr11p15.5‐p15.1: (180001‐18120000) X3;17.94 Mb	46,XX, der(9)t(9;11)(p24;p15)pat	Pathogenic Pathogenic	True Positive
SJ047	Ventriculomegaly	chr1p36.32‐p36.23: (810001‐9010000) X1;8.2 Mb	chr1p36.33‐p36.23: (820001‐9140000) X1;8.32 Mb	46,XY,t(1;9)(p36;q12)	Pathogenic involved 1p36 microdeletion syndrome	True Positive
SJ101	VSD;AS	chr9p24.3‐p23: (310001‐12110000) X1;11.8 Mb	chr9p24.3‐p23: (200001‐12940000) X1;12.74 Mb	Unavailable	Pathogenic	True Positive
SJ001	Cystic hydroma; hydroderma	chrYp11.3‐p11.1: (1000‐11400000) X1;11.39 Mb	chrYp11.3‐p11.1: (1‐11600000) X1;11.6 Mb	Unavailable	Pathogenic	True Positive
SJ022	ASD	chr22q11.1‐q11.21: (17400000‐21499999) X3;4.1 Mb	chr22q11.1‐q11.21: (16840001‐21460000) X3;4.62 Mb	47,XY,+mar	Pathogenic involved 22q11 duplication syndrome	True Positive
SJ103	TOF	chr17p13.3‐p13.2:（10000‐3530000); X1;3.52 Mb	chr17p13.3‐p13.2:（1‐3400000); X1;3.4 Mb	46,XX	Pathogenic involved Miller‐Dieker syndrome (MDS)	True Positive

Abbreviations: AS, aortic stenosis; ASD, atrial septal defect; CNVs, copy number variations; CNV‐seq, copy number variation sequencing; Mb, Megabyte; NIPT, non‐invasive prenatal testing; SUA, single umbilical artery; TOF, tetralogy of Fallot; VSD, ventricular septal defect.

**Table 2:** The performance of NIPT for CNVs detection

CNVs size	TP	FP	FPR%	PPV%	Sensitivity%	TN	FN	FNR%	NPV%	Specificity%
1 Mb ≤ CNVs <5 Mb	3	0	0	100	100	158	0	0	100	100
CNVs ≥ 5 Mb	7	1	0.65	87.5	77.78	152	2	22.22	98.70	99.35
CNVs ≥ 1 Mb	10	1	0.66	90.91	83.33	150	2	16.67	98.68	99.34

Abbreviations: CNVs, copy number variations; FN, false negative; FNR, false negative rate; FP, false positive; FPR, false positive rate; NPV, negative predictive rate; PPV, positive predictive rate; TN, true negative; TP, true positive.

## 4 DISCUSSION

Cell‐free foetal DNA (cffDNA) can definitely lead to occasional discordant NIPT results as the detection target of NIPT. Increasing evidences have shown that cffDNA in maternal plasma is derived predominantly from placental trophoblastic cells,^13^ so NIPT reflects the genetic information of the placenta, not foetus.^14^, ^15^, ^16^ Confined placental mosaicism (CPM) indicates that chromosome aberrations only exist in placenta and not in foetus, which is widely accepted as a cause of false positive NIPT results.^17^ Confined placental mosaicism is also reportedly relevant to intrauterine growth restriction (IUGR) and an increased risk of perinatal morbidity and mortality.^18^ Coincidentally, the later ultrasound findings in case SJ023 showed IUGR, which added evidence to our speculation. Maternal background is believed as another contributing factor to affect NIPT performance. In this case, maternal interference was excluded by maternal DNA sequencing. Placental mosaicism may have led to the false negative result in case SJ015, which meant that the chromosomal constitutions of the foetus and placenta were both abnormal. The degree of mosaicism could vary greatly in different regions of placental tissue. If more cffDNA from the lower level mosaic region was released into the maternal plasma, the NIPT result would be negative.^19^ As a biological factor, the mosaicism either in foetus or in placenta, is a limitation of NIPT accuracy that cannot be overcome.^11^ CPM and placental mosaicism were usually used to explain the discordant result of detecting aneuploidy in previous studies, our theoretical speculation was hard to be confirmed due to the unavailable placental tissue. Further research will focus on finding the difference in the chromosomal constitutions of the foetus, placenta and the different placental tissue regions.

Low foetal fraction is a common reason for false negative NIPT results.^20^ However, it cannot explain the false negative result in case SJ028, where the foetal fraction of 17.77% was higher than the detection threshold of 4%. We speculated that the false negative result may have been associated with the position of the CNVs. In maternal plasma, the sizes, GC contents and measurement coefficient of variances (CVs) of the cffDNA fragments from various chromosome locations may vary largely, due to the different degradation rates among individuals.^21^, ^22^ Therefore, the NIPT sequencing data analysis would be affected correspondingly, especially for CNVs near the telomeres and centromeres.^8^ With current techniques, NIPT cannot exhibit high performance in detecting genome‐wide CNVs, but in a selected few chromosome aberrations, such as 22q11.2 microdeletion.^11^ In our study, NIPT showed higher performance in detecting smaller sizes CNVs than previous studies,^7^, ^8^ demonstrating the improved technology of NIPT was independent with CNVs size.

It was recommended that pregnancies with foetal structural anomalies should undergo invasive procedures for diagnosing CNVs, rather than non‐invasive screening. However, our study showed that the residue risk of chromosome abnormalities was still remained in the pregnancies with isolated softmarkers, and even a few foetuses with pathogenic CNVs may have normal ultrasound findings.^23^ Copy number variations were also diagnosed in the foetuses with parental derivative chromosomes in our study, which may be misdiagnosed by negative NIPT results for aneuploidy, if the family history was unknown in advance. In addition, unlike Down's syndrome, the risk of pathogenic CNVs is not closely related to maternal age, and even younger women are liable to suffer from microdeletion in foetus.^24^ If the detection range is expanded from aneuploidy to CNVs in first trimester, the detection rate of chromosome abnormalities will be higher, and the benefit population will be wider, especially for the pregnancies without indications for invasive procedures. Basing on the above demonstrations, we think that NIPT could be performed on the pregnant women with foetal ultrasound abnormalities for CNVs detection, and it could be expanded to the pregnant women with other high‐risk factors. Moreover, NIPT for CNVs detection in a general risk group will need further evaluation in future studies, with the improvements of NIPT technology and the accumulation of relevant data about the pathogenicities of CNVs.

NIPT can be a better method of screening for chromosomal aberrations in first trimester. When cffDNA is used as the detection target, the following intrinsic drawbacks cannot be solved: the discordant caused by CPM or placental mosaicism, maternal CNVs interference, low foetal fraction, various degradation rates of cffDNA in maternal plasma and the uncertain accuracy of detecting twins or multiple pregnancies. So people have been looking for other new targets, and foetal nucleated red blood cells (fNRBCs) have attached much attention. Future efforts are made to develop new non‐invasive prenatal testing methods.

## CONFLICT OF INTEREST

The authors declare that they have no conflicts of interest.

## AUTHOR CONTRIBUTION

Wanting Cui: performed the research and drafted the manuscript; Xiaoliang Liu and Rong He: collected the samples and offered genetic counselling; Yuanyuan Zhang, Yueping Wang and Guoming Chu: analysed the data and edited the manuscript; Yanyan Zhao: designed the research and edited the manuscript.
